# To warrant clinical adoption AI models require a multi-faceted implementation evaluation

**DOI:** 10.1038/s41746-024-01064-1

**Published:** 2024-03-06

**Authors:** Davy van de Sande, Eline Fung Fen Chung, Jacobien Oosterhoff, Jasper van Bommel, Diederik Gommers, Michel E. van Genderen

**Affiliations:** 1 Erasmus MC University Medical Center, Department of Adult Intensive Care, Rotterdam, The Netherlands; 2 Delft University of Technology, Faculty of Technology, Policy and Management, Delft, The Netherlands

**Keywords:** Health care, Medical ethics

## Abstract

Despite artificial intelligence (AI) technology progresses at unprecedented rate, our ability to translate these advancements into clinical value and adoption at the bedside remains comparatively limited. This paper reviews the current use of implementation outcomes in randomized controlled trials evaluating AI-based clinical decision support and found limited adoption. To advance trust and clinical adoption of AI, there is a need to bridge the gap between traditional quantitative metrics and implementation outcomes to better grasp the reasons behind the success or failure of AI systems and improve their translation into clinical value.

## Current AI evaluation in healthcare

Despite the increasing interest in using artificial intelligence (AI) for healthcare to improve clinical decision making and patient outcomes, less than 2% of AI models reach beyond the prototyping phase and the actual clinical value of AI at the bedside remains largely unknown^1^. Examples of such AI-based clinical decision support systems (AICDSS) include those used to predict the administration of fluids or vasopressors in sepsis patients admitted to the intensive care unit (ICU)^2^ or electrocardiogram analysis to diagnose a myocardial infarction^3^. To effectively determine the clinical value of an AICDSS, these would ideally be evaluated through a randomized controlled trial (RCT), comparing it against the gold standard or placebo^4^.

Currently, only a limited amount of such RCT’s have been conducted. Most of them are analyzed in two systematic reviews evaluating the effectiveness of AICDSS in real-world clinical settings^5,6^. Although these trials demonstrated promising statistical AI performance, almost half of them did not show improved patient outcomes. Consider the scenario of an AICDSS for sepsis prediction in ICUs. Despite its high statistical accuracy, its clinical adoption is limited due to extensive data verification demands causing workflow disruption, and potentially delaying treatment, and concerns regarding the AI’s decision-making process and its transparency hindering the full acceptance and adoption in clinical practice. These factors lead to hesitation among ICU professionals, underlining that quantitative metrics like AUROC and accuracy are insufficient to evaluate AI in clinical settings, as they not accurately reflect the real-world utility.

To ensure a comprehensive understanding of AI’s functionality in clinical practice, it’s essential to employ a multi-faceted evaluation approach^7^. This includes, for example, the mixed-methods approach proposed by Smith et al.^8^. Despite acknowledging the importance of human-AI interaction, current guidelines such as DECIDE-AI^9^ and CONSORT-AI^10^ fail to offer adequate measures for evaluating the success of implementing an AI. Therefore, we analyzed the RCTs identified in the two largest systematic reviews on AI-based RCTs to date^5,6^ and categorized their outcomes according to a well-established taxonomy of implementation outcomes developed by Proctor et al.^11^. This taxonomy includes eight key implementation outcomes: acceptability, appropriateness, feasibility, fidelity, adoption, penetration, implementation cost and sustainability (definitions can be found in Supplementary Table 1).

**Fig. 1:**
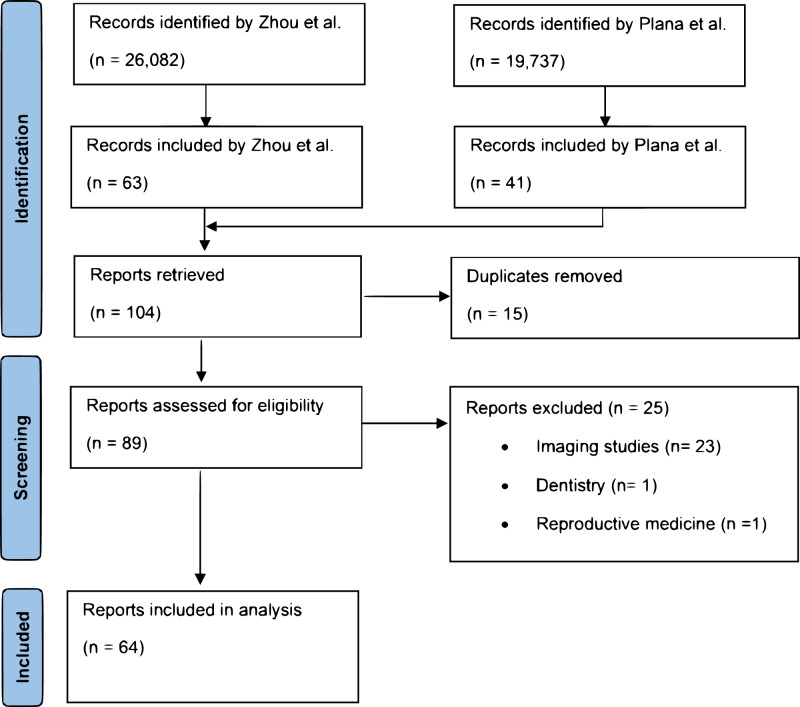
Flow diagram of the study review process and the exclusion of studies. Randomized controlled trials evaluating the effectiveness of artificial intelligence-based clinical decision support systems in real-world clinical settings were extracted from previous systematic reviews by Zhou et al.^5^ and Plana et al.^6^.

We thoroughly analyzed a total of 104 RCTs, extracted from the two systematic reviews; one by Zhou et al.^5^ (yielding 63 studies from an initial pool of 26,082) and one by Plana et al.^6^ (yielding 41 studies from 19,737 identified). The review process was conducted by the following authors (DvdS, EFFC, MvG), see Fig. 1. Duplicates (*n* = 15) were removed and imaging studies (*n* = 23), studies in dentistry (*n* = 1), and reproductive medicine (*n* = 1) were excluded. The taxonomy of implementation outcomes was applied independently by three authors (DvdS, EFFC, MvG). Discrepancies in the final classification were resolved by discussion with another panel member (JO). For each RCT, we meticulously extracted and recorded the author, year of publication, country and reported implementation outcomes (according to definitions described in Supplementary Table 1).

64 RCTs were included in the analysis, see Supplementary Table 2. Among these, 31 RCTs (48%) did not report significant improvement of the primary outcome, while 24 RCTs (38%) did not report on implementation outcomes. Additionally, 21 RCTs (33%) reported two or more implementation outcomes. The most frequently reported implementation outcome was ‘Fidelity’ (31 RCTs [48%]) which refers to ‘*the degree to which the AI system was implemented as it was prescribed in the original protocol or as it was intended by the system manufacturer’*. On the other hand, ‘Adoption’ (*the intention, initial decision, or action to try or employ the AI system*) (6 RCTs [9%]), ‘Appropriateness’ (*the perceived fit, clinical relevance, or compatibility of the AI system for a specific clinical setting, provider, or consumer; and/or perceived fit of the innovation to address a particular clinical problem*), ‘Implementation costs’ (*the cost impact of the AI system in a given clinical setting)*, ‘Sustainability’ (*the extent to which a newly implemented AI system is maintained or institutionalized within a service setting’s ongoing, stable operations*), and ‘Penetration’ (*the integration of the AI system in the clinical workflow and its subsystems*) were reported in less than 10% of the RCTs (see Table 1).

**Table 1:** Implementation outcomes reported in randomized controlled trials studying artificial intelligence in healthcare

			Reported in *N* (%)	
Implementation outcome^a^	Clinical explanation	Implementation stage	RCTs (*N* = 64)	Guidelines^b^ (*N* = 5)
Appropriateness	Is the AI compatible with the clinical workflow and is it useful?	Early	5 (8)	0 (0)
Acceptability	Is the AI acceptable, agreeable, or satisfactory for the users?	Ongoing	10 (16)	0 (0)
Feasibility	Can the AI be successfully used as intended by the manufacturer?	Early	16 (25)	0 (0)
Adoption	Do the users express the initial decision, or action to try or employ the AI?	Ongoing	6 (9)	0 (0)
Fidelity	Is the AI implemented as intended by the manufacturer?	Ongoing	31 (48)	0 (0)
Implementation cost	What is the cost impact of implementing the AI system?	Late	4 (6)	0 (0)
Penetration	Has the AI been adopted by all groups of trained users?	Late	0 (0)	0 (0)
Sustainability	Is the AI maintained within ongoing clinical operations over time?	Late	1 (2)	0 (0)

*AI* artificial intelligence, *RCTs* randomized controlled trials.

^a^Definitions of implementation outcomes were adapted from the taxonomy of implementation outcomes by Proctor et al.^11^.

^b^AI specific guidelines: TRIPOD-AI and STARD-AI, DECIDE-AI, SPIRIT-AI, CONSORT-AI.

Our analysis reveals that while the number of RCTs conducted in hospital settings (31 studies, 48%) and non-hospital settings (33 studies, 52%) was similar, a notable disparity emerges in the reporting of implementation outcomes. Specifically, RCTs in non-hospital settings reported these outcomes more frequently (42%, 14 of 33 studies) compared to those in hospital settings (23%, 7 of 31 studies). This might suggest that the complexity or constraints of hospital environments pose challenges in evaluating and reporting these outcomes. Furthermore, when examining the AI interventions’ objectives, we found that RCTs assessing AICDSS for lifestyle management or self-care improvement were more likely to consider multiple implementation outcomes (64%, 7 out of 11 studies). In contrast, those focusing on treatment support or diagnostic improvement were less likely to do so, with 33% (8 out of 24 studies) and 22% (2 out of 9 studies), respectively. These findings underscore the importance of considering the setting and nature of AI interventions when designing and reporting RCTs.

Currently, the majority of RCTs evaluating AICDSS in real clinical settings lack comprehensive evaluation of outcomes essential for understanding implementation success. The most commonly reported implementation outcome (‘fidelity’), continued to be measured using quantitative metrics, while outcomes such as ‘acceptability’ and ‘appropriateness’ that require a qualitative approach are given less attention, potentially due to the additional time involved. Our findings show that only 33% of the RCTs comprehensively evaluate multiple aspects of AICDSS implementation, reflecting a gap in understanding the broader impact of AICDSS implementation. This limitation is further exacerbated by the possibility that some RCTs may collect implementation data but fail to include it in their published reports. Despite this it is crucial for the clinical AI field to not only confirm the effectiveness of AICDSS but also to grasp the contextual factors that drive its success or failure. In line with this, we advocate for additional systematic reporting of implementation outcomes, in addition to effectiveness metrics and standard reporting items, as recommended by the CONSORT-AI and DECIDE-AI guidelines.

## Future AI evaluation in healthcare

**Fig. 2:**
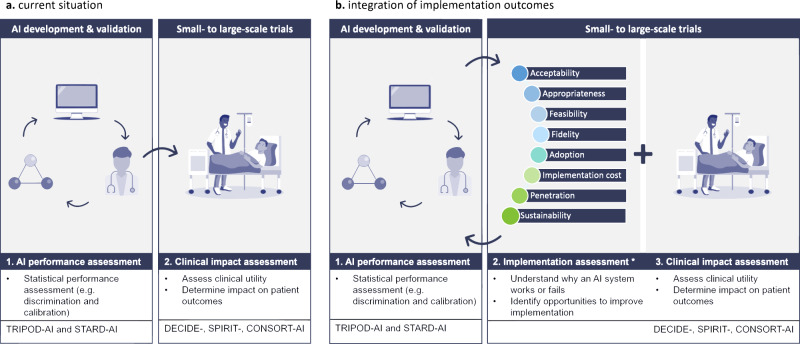
Overview of the current and desired approach to evaluate artificial intelligence in healthcare. **a** In the current situation, artificial intelligence-based clinical decision support systems (AI-CDSS), are clinically deployed, after going through multiple preclinical validations (e.g., external and temporal algorithm validation) to assess their clinical utility and effectiveness. **b** To enhance comprehension of factors that contributed to successful implementation or failure at the bedside, implementation outcomes should be systematically integrated in future clinical trials evaluating AICDSS in real-world clinical settings. *Implementation outcomes as described by Proctor et al.^11^.

It is our moral obligation to ensure trustworthy and responsible adoption of AI in healthcare^12^. Although current guidelines improve transparent reporting of technical model development and deployment, they fall short in addressing the essential implementation processes that determine the actual clinical benefits. Additionally, the regulatory approval process for these devices primarily focuses on concerns related to safety, performance, and risk-benefit considerations, neglecting factors that influence the adoption of AI at the patient’s bedside. To establish transparency and foster trust among healthcare professionals and, ultimately, patients, it is vital to develop a comprehensive understanding of the factors that contribute to both successful and unsuccessful implementations in clinical settings^8^. Common barriers that hinder clinical adoption of AI, include concerns about loss of autonomy, limited integration into clinical workflows, reduced patient interaction, unintuitive user interface, and unreliable internet connections^13^. These obstacles cannot be adequately captured through quantitative measures alone; they require a multi-faceted approach. Consequently, it is necessary to expand the implementation science of AI beyond current guidelines towards an approach that incorporates the evaluation of implementation outcomes alongside measures of effectiveness in future clinical trials (see Fig. 2). By evaluating these outcomes, we can not only evaluate the statistical performance of the AI but also assess the adoption, usability, and real-world impact of clinical AI-based interventions in healthcare settings. Additionally, this approach allows us to identify barriers, facilitators, and strategies for enhancing and sustaining these interventions over time.

To continuously evaluate AI’s impact in healthcare in the form of a framework and develop strategies for its ethical integration, a multi-faceted approach is essential. This involves regular and systematic assessment of the AI implementation process in both RCTs and clinical practice. While implementation outcomes are valuable for measuring the success or failure of AI implementation, they require in-depth analysis within well-established frameworks like the Consolidated Framework for Implementation Research (CFIR)^14^ to fully understand the implications. Additionally, models like the Unified Theory of Acceptance and Use of Technology (UTAUT)^15^ offer valuable insights into factors influencing the users’ acceptance of AI within a clinical setting. For a comprehensive assessment of implementation outcomes, we recommend establishing robust, semiquantitative, and reproducible evaluation tools, which can be adapted from validated resources such as those in the Implementation Outcome Repository launched by the National Institute for Health Research Applied Research Collaboration South London^16^. The AI implementation process in AICDSS should undergo continuous measurement and adjustment. This involves designing specific implementation strategies for the AICDSS, identify key outcomes for ongoing evaluation of success or failure, and evaluate barriers and facilitators for implementation using frameworks such as CFIR. A structured Plan-Do-Study-Act cycle^17^ should be followed, with a predetermined timeframe for evaluation. Based on the outcomes, the AI implementation strategy is adjusted as needed, ensuring a dynamic and responsive approach for sustained success (Fig. 2).

Traditional RCTs, despite their methodological strengths, may not always be the most suitable approach for exploring the complex dynamics of AI implementation in clinical settings. Therefore, a RCT with a dual focus on assessing both clinical effectiveness and implementation, known as ‘Effectiveness-implementation hybrid design’, offers a more nuanced methodology^18^. In practical terms, this involves designing an RCT that not only measures clinical outcomes but also gathers predefined information on implementation outcomes and implementation factors.

## Conclusion

Ultimately, we have to bridge the gap between technological AI model development and trustworthy and responsible AI that is being adopted in a clinical setting. As such it is imperative to systematically incorporate implementation outcomes throughout the entire so called ‘clinical AI lifecycle’. This integration should begin during the early stages of preclinical development and continue as a priority when implementing AI at the patients’ bedside. By expanding the field of implementation science in AI, we can successfully transform AI predictions into optimal human interventions. This is essential to trust AI and unlock its full potential in revolutionizing healthcare delivery and improving patient outcomes.

### Reporting summary

Further information on research design is available in the Nature Research Reporting Summary linked to this article.

### Supplementary information

Supplementary Information

Reporting Summary

## Data Availability

The dataset analyzed during the current study is available from the corresponding author on request.
