# Supplementary material for: To warrant clinical adoption AI models require a multi-faceted implementation evaluation
Source: NPJ Digit Med. 2024 Mar 6;7:58. doi: 10.1038/s41746-024-01064-1 (PMC10918103; doi:10.1038/s41746-024-01064-1)
Supplement: Supplementary file 1 — Supplementary Information [file 41746_2024_1064_MOESM1_ESM.pdf]

Supplementary Information

Title: To warrant clinical adoption AI models require a multi-faceted implementation evaluation

Authors: Davy van de Sande Bsc[1], Eline Fung Fen Chung Msc[1], Jacobien Oosterhoff MD PhD[2], Jasper van Bommel MD PhD[1], Diederik Gommers MD PhD[1],

Michel E. van Genderen MD PhD[1]

1. Erasmus MC University Medical Center, Department of Adult Intensive Care, Rotterdam, The Netherlands
2. Delft University of Technology, Faculty of Technology, Policy and Management, Delft, The Netherlands

Corresponding author:

Michel E. van Genderen

Erasmus MC University Medical Center, Rotterdam, Netherlands

m.vangenderen@erasmusmc.nl

Supplementary Table 1: Implementations outcomes and corresponding definitions.

Implementation outcome	Definition*
Acceptability	This implementation outcome refers to how agreeable, palatable, or satisfactory an AI intervention is perceived. Lack of acceptability has been noted as a challenge in AI implementation. The referent of the implementation outcome 'acceptability' (or the 'what' is acceptable) may be a specific AI intervention or service within a particular setting. Acceptability should be assessed based on the stakeholder's knowledge of or direct experience with various dimensions of the AI to be implemented, such as its content, complexity, or comfort. Acceptability may be measured from the perspective of various stakeholders, such as administrators, payers, providers, and consumers.
Adoption	This implementation outcome is centered around the intention, initial decision, or action to try or employ an AI system, often termed to as 'uptake.' It specifically relates to the number of participants or users who employ the AI, irrespective of quality or adherence to the protocol for clinical use.
Appropriateness	The perceived fit, relevance, or compatibility of the AI for a given clinical setting, user, or consumer, and/or the perceived fit of the AI to address a specific clinical problem. The construct 'appropriateness' is deemed important for its potential to capture some 'pushback' to AI implementation efforts, as is seen when users feel a new AI is a 'stretch' from the mission of the clinical setting or is not consistent with users' skill set, role, or job expectations.
Implementation cost	Cost (Incremental or implementation cost) is defined as the cost impact of clinically implementing an AI implementation. AI implementation costs vary depending on complexity of the AI itself, chosen implementation strategy, type of clinical setting in which the AI is implemented; not to be confused with benefit or efficiency.

Feasibility	Defined as the extent to which a new AI can be successfully used or carried out within a given clinical setting. Typically, the concept of feasibility is invoked retrospectively as a potential explanation of an AI's success or failure, as reflected in poor AI integration, usage, or participation rates.
Fidelity	Defined as the degree to which an AI was implemented as it was prescribed in accordance with its original protocol or as it was intended by its developers. It encompassess aspects such as adherence, quality of AI delivery, program component differentiation, exposure to the AI intervention, and user responsiveness or involvement. Ensuring high fidelity is crucial for evaluating the AI's effectiveness as intended.
Penetration	Refers to the extent of integration of an AI system within a clinical setting and its subsystems. It can also be quantified as the number of healthcare providers who actively use an AI, divided by the total number of healthcare providers trained in or expected to use the AI.
Sustainability	Defined as the extent to which a newly implemented AI is maintained or institutionalized within routine clinical practice.

AI= Artificial Intelligence.

*Definitions were modified from Proctor et al.[1] to better fit the clinical Artificial Intelligence setting.

Supplementary Table 2: List of all included studies and identified implementation outcomes.

Lead Author	Country lead author	Year	Clinical domain	Aim of intervention categorized	Number of implementation outcomes	Acceptability	Adoption	Appropriateness	Implementation cost	Feasibility	Fidelity	Penetration	Sustainability	Effect on primary outcome
Avari et al.[2]	United Kingdom	2021	General Internal Medicine	Decision support/treatment recommendations	2	☹	☹	☹	☹	☺	☺	☹	☹	☹
Bailey et al.[3]	United States	2013	General Internal Medicine	Patient deterioration prediction	0	☹	☹	☹	☹	☹	☹	☹	☹	☹
Blomberg et al.[4]	Denmark	2021	Emergency medicine	Improving diagnosis	1	☹	☹	☹	☹	☺	☹	☹	☹	☹
Brier et al.[5]	United States	2010	Nephrology	Decision support/treatment recommendations	1	☹	☹	☹	☹	☹	☺	☹	☹	☺
Brocklehurst et al.[6]	United Kingdom	2017	Gyneacology	Patient deterioration prediction	0	☹	☹	☹	☹	☹	☹	☹	☹	☹
Caballero-Ruiz et al.[7]	Spain	2017	General Internal Medicine	Lifestyle management and self-care support	3	☺	☹	☺	☹	☺	☺	☹	☹	☺
Caparros-Gonzalez et al.[8]	Spain	2018	Intensive Care	Stress reduction	0	☹	☹	☹	☹	☹	☹	☹	☹	☹
Clemons et al.[9]	Canada	2016	Oncology	Patient deterioration prediction	0	☹	☹	☹	☹	☹	☹	☹	☹	☺
Cox et al.[10]	United States	2019	Intensive Care	Prognostication	0	☹	☹	☹	☹	☹	☹	☹	☹	☹
El Solh et al.[11]	United States	2009	Sleep medicine	Decision support/treatment recommendations	0	☹	☹	☹	☹	☹	☹	☹	☹	☺

Finkelstein et al.[12]	United States	2013	Pulmonology	Improving diagnosis	2	☹	☹	☹	☹	☺	☺	☹	☹	☹
Forman et al.[13]	United States	2019	Obstetrics	Lifestyle management and self-care support	2	☺	☹	☹	☹	☹	☺	☹	☹	☺
Geersing et al.[14]	Netherlands	2020	Primary Care	Prognostication	1	☹	☹	☹	☹	☹	☺	☹	☹	☹
Guenancia et al.[15]	France	2016	Cardiology	Prognostication	1	☹	☹	☹	☹	☹	☺	☹	☹	☹
Hill et al.[16]	United Kingdom	2011	Primary Care	Decision support/treatment recommendations	1	☹	☹	☹	☺	☹	☹	☹	☹	☺
Hsu et al.[17]	Taiwan	2013	Intensive Care	Decision support/treatment recommendations	0	☹	☹	☹	☹	☹	☹	☹	☹	☺
Jayakumar et al.[18]	United States	2021	Orthopedics	Decision support/treatment recommendations	1	☹	☹	☹	☹	☺	☹	☹	☹	☺
Kappen et al.[19]	Netherlands	2014	Anesthesiology	Decision support/treatment recommendations	3	☺	☹	☹	☺	☺	☹	☹	☹	☹
Kougias et al.[20]	United States	2019	Surgery	Workflow optimization	0	☹	☹	☹	☹	☹	☹	☹	☹	☺
Lauffenburger et al.[21]	United States	2019	Primary Care	Prognostication	1	☹	☹	☹	☹	☹	☺	☹	☹	☹
Lustrek et al.[22]	Slovenia	2021	Cardiology	Lifestyle management and self-care support	2	☺	☹	☹	☹	☹	☺	☹	☹	☺
Mahler et al.[23]	United States	2015	Emergency medicine	Decision support/treatment recommendations	1	☹	☹	☹	☹	☹	☺	☹	☹	☺
Mán et al.[24]	Hungary	2014	Surgery	Decision support/treatment	1	☹	☹	☹	☹	☹	☺	☹	☹	☹

				recommendations										
Mann et al.[25]	United States	2020	Primary Care	Decision support/treatment recommendations	3	☹	☺	☹	☹	☹	☺	☹	☺	☹
Manz et al.[26]	United States	2020	Oncology	Prognostication	0	☹	☹	☹	☹	☹	☹	☹	☹	☺
Martin et al.[27]	Ireland	2012	Primary Care	Disease prevention	1	☹	☹	☹	☹	☹	☺	☹	☹	☺
Mazurek et al.[28]	United States	2020	Primary Care	Lifestyle management and self-care support	2	☹	☺	☹	☹	☹	☺	☹	☹	☹
McGinn et al.[29]	United States	2013	Primary Care	Decision support/treatment recommendations	2	☹	☺	☹	☹	☹	☺	☹	☹	☺
Meijer et al.[30]	Netherlands	2020	Anesthesiology	Decision support/treatment recommendations	0	☹	☹	☹	☹	☹	☹	☹	☹	☺
Nieuwlaat et al.[31]	Canada	2012	Primary Care	Decision support/treatment recommendations	2	☹	☹	☹	☹	☺	☺	☹	☹	☹
Nimri et al.[32]	Israel	2020	Endocrinology	Lifestyle management and self-care support	4	☺	☺	☺	☹	☺	☺	☹	☹	☺
Palen et al.[33]	United States	2019	Primary Care	Decision support/treatment recommendations	1	☹	☹	☹	☹	☹	☺	☹	☹	☺
Pavel et al.[34]	Ireland	2020	Neonatology	Improving diagnosis	0	☹	☹	☹	☹	☹	☹	☹	☹	☹
Persell et al.[35]	United States	2013	Primary Care	Decision support/treatment recommendations	1	☹	☹	☹	☹	☺	☹	☹	☹	☹

Persell et al.[36]	United States	2020	Primary Care	Lifestyle management and self-care support	1	☹	☹	☹	☹	☹	☺	☹	☹	☹
Pielmeier et al.[37]	Denmark	2012	Intensive Care	Decision support/treatment recommendations	0	☹	☹	☹	☹	☹	☹	☹	☹	☺
Plomb-Homes et al.[38]	Switzerland	2018	Rehabilitation	Prognostication	1	☺	☹	☹	☹	☹	☹	☹	☹	☹
Poldervaart et al.[39]	Netherlands	2017	Emergency medicine	Decision support/treatment recommendations	2	☹	☹	☹	☺	☹	☺	☹	☹	☹
Rabbi et al.[40]	United States	2015	Primary Care	Lifestyle management and self-care support	4	☺	☹	☺	☹	☺	☺	☹	☹	☺
Sadasivam et al.[41]	United States	2016	Primary Care	Lifestyle management and self-care support	2	☹	☹	☹	☹	☺	☺	☹	☹	☺
Sáenz et al.[42]	Spain	2012	Primary Care	Decision support/treatment recommendations	0	☹	☹	☹	☹	☹	☹	☹	☹	☹
Sandal et al.[43]	Denmark	2021	General Practice	Lifestyle management and self-care support	1	☹	☹	☹	☹	☹	☺	☹	☹	☺
Seol et al.[44]	United States	2021	Pediatrics	Decision support/treatment recommendations	0	☹	☹	☹	☹	☹	☹	☹	☹	☹
Sheridan et al.[45]	United States	2011	General Internal Medicine	Prognostication	2	☹	☹	☹	☹	☺	☺	☹	☹	☺
Sherratt et al.[46]	United Kingdom	2016	Primary Care	Prognostication	0	☹	☹	☹	☹	☹	☹	☹	☹	☹
Shimabukuro et al.[47]	United States	2017	Intensive Care	Improving diagnosis	0	☹	☹	☹	☹	☹	☹	☹	☹	☺

Snooks et al.[48]	United Kingdom	2018	Primary Care	Prognostication	4	☺	☺	☹	☺	☺	☹	☹	☹	☺
Steiner et al.[49]	United States	2016	Primary Care	Workflow optimization	0	☹	☹	☹	☹	☹	☹	☹	☹	☺
Steiner et al.[50]	United States	2018	Primary Care	Workflow optimization	2	☺	☹	☹	☹	☺	☹	☹	☹	☺
Steinhart et al.[51]	Canada	2016	Emergency medicine	Improving diagnosis	0	☹	☹	☹	☹	☹	☹	☹	☹	☹
Stiell et al.[52]	Canada	2010	Emergency medicine	Decision support/treatment recommendations	2	☹	☺	☹	☹	☹	☺	☹	☹	☹
Strömblad et al.[53]	United States	2021	Surgery	Workflow optimization	0	☹	☹	☹	☹	☹	☹	☹	☹	☺
Tan et al.[54]	Singapore	2020	Surgery	Improving diagnosis	0	☹	☹	☹	☹	☹	☹	☹	☹	☹
Thurtle et al.[55]	United Kingdom	2019	Oncology	Prognostication	2	☺	☹	☺	☹	☹	☹	☹	☹	☺
Torres et al.[56]	Argentina	2014	Primary Care	Decision support/treatment recommendations	0	☹	☹	☹	☹	☹	☹	☹	☹	☺
True et al.[57]	United States	2015	Primary Care	Prognostication	0	☹	☹	☹	☹	☹	☹	☹	☹	☹
van de Maat et al.[58]	Netherlands	2020	Pediatrics	Decision support/treatment recommendations	2	☹	☹	☹	☹	☺	☺	☹	☹	☹
van Driest et al.[59]	United States	2020	Intensive Care	Improving diagnosis	1	☹	☹	☹	☹	☹	☺	☹	☹	☺
Vos-Kerkhof et al.[60]	Netherlands	2015	Emergency medicine	Decision support/treatment recommendations	1	☹	☹	☹	☹	☹	☺	☹	☹	☹
Voss et al.[61]	United States	2019	Pediatrics	Lifestyle management and self-care support	1	☹	☹	☹	☹	☹	☺	☹	☹	☺
Wang et al.[62]	United States	2019	Primary Care	Improving diagnosis	3	☹	☹	☺	☹	☺	☺	☹	☹	☹
Wijnberge et al.[63]	Netherlands	2020	Anesthesiology	Decision support/treatm	1	☹	☹	☹	☹	☹	☺	☹	☹	☺

				ent recommendations										
Yao et al.[64]	United States	2021	Cardiology	Improving diagnosis	0	☹	☹	☹	☹	☹	☹	☹	☹	☺
Zeevi et al.[65]	Israel	2015	Primary Care	Lifestyle management and self-care support	0	☹	☹	☹	☹	☹	☹	☹	☹	☹

☺= Outcomes is reported; ☹= Outcome is not reported.

Supplementary References

1. Proctor, E., *et al.* Outcomes for implementation research: conceptual distinctions, measurement challenges, and research agenda. *Adm Policy Ment Health* **38**, 65-76 (2011).
2. Avari, P., *et al.* Safety and Feasibility of the PEPPER Adaptive Bolus Advisor and Safety System: A Randomized Control Study. *Diabetes Technol The* **23**, 175-186 (2021).
3. Bailey, T.C., *et al.* A trial of a real-time alert for clinical deterioration in patients hospitalized on general medical wards. *J Hosp Med* **8**, 236-242 (2013).
4. Blomberg, S.N., *et al.* Effect of Machine Learning on Dispatcher Recognition of Out-of-Hospital Cardiac Arrest During Calls to Emergency Medical Services A Randomized Clinical Trial. *Jama Network Open* **4**(2021).
5. Brier, M.E., Gaweda, A.E., Dailey, A., Aronoff, G.R. & Jacobs, A.A. Randomized trial of model predictive control for improved anemia management. *Clin J Am Soc Nephrol* **5**, 814-820 (2010).
6. Brocklehurst, P., *et al.* Computerised interpretation of fetal heart rate during labour (INFANT): a randomised controlled trial. *Lancet* **389**, 1719-1729 (2017).
7. Caballero-Ruiz, E., *et al.* A web-based clinical decision support system for gestational diabetes: Automatic diet prescription and detection of insulin needs. *Int J Med Inform* **102**, 35-49 (2017).
8. Caparros-Gonzalez, R.A., de la Torre-Luque, A., Diaz-Piedra, C., Vico, F.J. & Buela-Casal, G. Listening to Relaxing Music Improves Physiological Responses in Premature Infants A Randomized Controlled Trial. *Adv Neonat Care* **18**, 58-69 (2018).
9. Clemons, M., *et al.* Risk Model-Guided Antiemetic Prophylaxis vs Physician's Choice in Patients Receiving Chemotherapy for Early-Stage Breast Cancer A Randomized Clinical Trial. *Jama Oncol* **2**, 225-231 (2016).
10. Cox, C.E., *et al.* Effects of a Personalized Web-Based Decision Aid for Surrogate Decision Makers of Patients With Prolonged Mechanical Ventilation A Randomized Clinical Trial. *Ann Intern Med* **170**, 285-+ (2019).
11. El Solh, A.A., Akinnusi, M., Patel, A., Bhat, A. & TenBrock, R. Predicting optimal CPAP by neural network reduces titration failure: a randomized study. *Sleep Breath* **13**, 325-330 (2009).
12. Finkelstein, S.M., *et al.* A randomized controlled trial comparing health and quality of life of lung transplant recipients following nurse and computer-based triage utilizing home spirometry monitoring. *Telemed J E Health* **19**, 897-903 (2013).
13. Forman, E.M., *et al.* Randomized controlled trial of OnTrack, a just-in-time adaptive intervention designed to enhance weight loss. *Transl Behav Med* **9**, 989-1001 (2019).
14. Geersing, G.J., *et al.* Effect of tailoring anticoagulant treatment duration by applying a recurrence risk prediction model in patients with venous thromboembolism compared to usual care: A randomized controlled trial. *PLoS Med* **17**, e1003142 (2020).
15. Guenancia, C., *et al.* Clinical effectiveness of the systematic use of the GRACE scoring system (in addition to clinical assessment) for ischaemic outcomes and bleeding complications in the management of NSTEMI compared with clinical assessment alone: a prospective study. *Heart Vessels* **31**, 897-906 (2016).
16. Hill, J.C., *et al.* Comparison of stratified primary care management for low back pain with current best practice (STarT Back): a randomised controlled trial. *Lancet* **378**, 1560-1571 (2011).

17. Hsu, J.C., *et al.* Clinical verification of a clinical decision support system for ventilator weaning. *Biomed Eng Online* **12 Suppl 1**, S4 (2013).
18. Jayakumar, P., *et al.* Comparison of an Artificial Intelligence-Enabled Patient Decision Aid vs Educational Material on Decision Quality, Shared Decision-Making, Patient Experience, and Functional Outcomes in Adults With Knee Osteoarthritis A Randomized Clinical Trial. *Jama Netw Open* **4**(2021).
19. Kappen, T.H., *et al.* Impact of risk assessments on prophylactic antiemetic prescription and the incidence of postoperative nausea and vomiting: a cluster-randomized trial. *Anesthesiology* **120**, 343-354 (2014).
20. Kougias, P., *et al.* A Statistical Model-driven Surgical Case Scheduling System Improves Multiple Measures of Operative Suite Efficiency Findings From a Single-center, Randomized Controlled Trial. *Ann Surg* **270**, 1000-1004 (2019).
21. Lauffenburger, J.C., *et al.* Effectiveness of Targeted Insulin-Adherence Interventions for Glycemic Control Using Predictive Analytics Among Patients With Type 2 Diabetes A Randomized Clinical Trial. *Jama Network Open* **2**(2019).
22. Lugtrek, M., *et al.* A Personal Health System for Self-Management of Congestive Heart Failure (HeartMan): Development, Technical Evaluation, and Proof-of-Concept Randomized Controlled Trial. *Jmir Med Inf* **9**(2021).
23. Mahler, S.A., *et al.* The HEART Pathway Randomized Trial Identifying Emergency Department Patients With Acute Chest Pain for Early Discharge. *Circ-Cardiovasc Qual* **8**, 195-+ (2015).
24. Man, E., Simonka, Z., Varga, A., Rarosi, F. & Lazar, G. Impact of the Alvarado score on the diagnosis of acute appendicitis: comparing clinical judgment, Alvarado score, and a new modified score in suspected appendicitis: a prospective, randomized clinical trial. *Surg Endosc* **28**, 2398-2405 (2014).
25. Mann, D., *et al.* Impact of Clinical Decision Support on Antibiotic Prescribing for Acute Respiratory Infections: a Cluster Randomized Implementation Trial. *J Gen Intern Med* **35**, 788-795 (2020).
26. Manz, C.R., *et al.* Effect of Integrating Machine Learning Mortality Estimates With Behavioral Nudges to Clinicians on Serious Illness Conversations Among Patients With Cancer: A Stepped-Wedge Cluster Randomized Clinical Trial. *JAMA Oncol* **6**, e204759 (2020).
27. Martin, C.M., *et al.* Implementation of complex adaptive chronic care: the Patient Journey Record system (PaJR). *Journal of Evaluation in Clinical Practice* **18**, 1226-1234 (2012).
28. Mazurek, M.O., *et al.* Effectiveness of the Extension for Community Health Outcomes Model as Applied to Primary Care for Autism A Partial Stepped-Wedge Randomized Clinical Trial. *Jama Pediatr* **174**(2020).
29. McGinn, T.G., *et al.* Efficacy of an evidence-based clinical decision support in primary care practices: a randomized clinical trial. *JAMA Intern Med* **173**, 1584-1591 (2013).
30. Meijer, F., *et al.* Reduced postoperative pain using Nociception Level-guided fentanyl dosing during sevoflurane anaesthesia: a randomised controlled trial. *Brit J Anaesth* **125**, 1070-1078 (2020).
31. Nieuwlaat, R., *et al.* Randomised comparison of a simple warfarin dosing algorithm versus a computerised anticoagulation management system for control of warfarin maintenance therapy. *Thromb Haemost* **108**, 1228-1235 (2012).
32. Nimri, R., *et al.* Insulin dose optimization using an automated artificial intelligence-based decision support system in youths with type 1 diabetes. *Nature Medicine* **26**(2020).

33. Palen, T.E., Sharpe, R.E., Shetterly, S.M. & Steiner, J.F. Randomized Clinical Trial of a Clinical Decision Support Tool for Improving the Appropriateness Scores for Ordering Imaging Studies in Primary and Specialty Care Ambulatory Clinics. *Am J Roentgenol* **213**, 1015-1020 (2019).
34. Pavel, A.M.*, et al.* A machine-learning algorithm for neonatal seizure recognition: a multicentre, randomised, controlled trial. *Lancet Child Adolesc* **4**, 740-749 (2020).
35. Persell, S.D., Lloyd-Jones, D.M., Friesema, E.M., Cooper, A.J. & Baker, D.W. Electronic health record-based patient identification and individualized mailed outreach for primary cardiovascular disease prevention: a cluster randomized trial. *J Gen Intern Med* **28**, 554-560 (2013).
36. Persell, S.D.*, et al.* Effect of Home Blood Pressure Monitoring via a Smartphone Hypertension Coaching Application or Tracking Application on Adults With Uncontrolled Hypertension A Randomized Clinical Trial. *Jama Network Open* **3**(2020).
37. Pielmeier, U., Rousing, M.L., Andreassen, S., Nielsen, B.S. & Haure, P. Decision support for optimized blood glucose control and nutrition in a neurotrauma intensive care unit: preliminary results of clinical advice and prediction accuracy of the Glucosafe system. *J Clin Monit Comput* **26**, 319-328 (2012).
38. Plomb-Holmes, C., Hilfiker, R., Legere, B. & Luthi, F. Impact of a non-return-to-work prognostic model (WORRK) on allocation to rehabilitation clinical pathways: A single centre parallel group randomised trial. *Plos One* **13**(2018).
39. Poldervaart, J.M.*, et al.* Effect of Using the HEART Score in Patients With Chest Pain in the Emergency Department A Stepped-Wedge, Cluster Randomized Trial. *Ann Intern Med* **166**, 689-+ (2017).
40. Rabbi, M., Pfammatter, A., Zhang, M., Spring, B. & Choudhury, T. Automated Personalized Feedback for Physical Activity and Dietary Behavior Change With Mobile Phones: A Randomized Controlled Trial on Adults. *Jmir Mhealth Uhealth* **3**(2015).
41. Sadasivam, R.S., Borglund, E.M., Adams, R., Marlin, B.M. & Houston, T.K. Impact of a Collective Intelligence Tailored Messaging System on Smoking Cessation: The Perspect Randomized Experiment. *J Med Internet Res* **18**(2016).
42. Saenz, A.*, et al.* Development and validation of a computer application to aid the physician's decision-making process at the start of and during treatment with insulin in type 2 diabetes: a randomized and controlled trial. *J Diabetes Sci Technol* **6**, 581-588 (2012).
43. Sandal, L.F.*, et al.* Effectiveness of App-Delivered, Tailored Self-management Support for Adults With Lower Back Pain-Related Disability A SELFBACK Randomized Clinical Trial. *Jama Internal Medicine* **181**, 1288-1296 (2021).
44. Seol, H.Y.*, et al.* Artificial intelligence-assisted clinical decision support for childhood asthma management: A randomized clinical trial. *Plos One* **16**(2021).
45. Sheridan, S.L.*, et al.* A randomized trial of an intervention to improve use and adherence to effective coronary heart disease prevention strategies. *Bmc Health Serv Res* **11**(2011).
46. Sherratt, F.C., Marcus, M.W., Robinson, J. & Field, J.K. Utilizing Lung Cancer Risk Prediction Models to Promote Smoking Cessation: Two Randomized Controlled Trials. *Am J Health Promot* **32**, 1196-1205 (2018).
47. Shimabukuro, D.W., Barton, C.W., Feldman, M.D., Mataraso, S.J. & Das, R. Effect of a machine learning-based severe sepsis prediction algorithm on patient survival and hospital length of stay: a randomised clinical trial. *Bmj Open Respir Res* **4**(2017).
48. Snooks, H.*, et al.* in *Predictive risk stratification model: a randomised stepped-wedge trial in primary care (PRISMATIC)* (Southampton (UK), 2018).

49. Steiner, J.F., Shainline, M.R., Bishop, M.C. & Xu, S. Reducing Missed Primary Care Appointments in a Learning Health System Two Randomized Trials and Validation of a Predictive Model. *Med Care* **54**, 689-696 (2016).
50. Steiner, J.F., Shainline, M.R., Dahlgren, J.Z., Kroll, A. & Xu, S. Optimizing Number and Timing of Appointment Reminders: A Randomized Trial. *Am J Manag Care* **24**, 377-384 (2018).
51. Steinhart, B.D.*, et al.* A Randomized Control Trial Using a Validated Prediction Model for Diagnosing Acute Heart Failure in Undifferentiated Dyspneic Emergency Department Patients-Results of the GASP4Ar Study. *J Card Fail* **23**, 145-152 (2017).
52. Stiell, I.G.*, et al.* A prospective cluster-randomized trial to implement the Canadian CT Head Rule in emergency departments. *CMAJ* **182**, 1527-1532 (2010).
53. Stromblad, C.T.*, et al.* Effect of a Predictive Model on Planned Surgical Duration Accuracy, Patient Wait Time, and Use of Presurgical Resources: A Randomized Clinical Trial. *Jama Surg* **156**, 315-321 (2021).
54. Tan, W.J.*, et al.* Randomized control trial comparing an Alvarado Score-based management algorithm and current best practice in the evaluation of suspected appendicitis. *World J Emerg Surg* **15**(2020).
55. Thurtle, D.R., Jenkins, V., Pharoah, P.D. & Gnanapragasam, V.J. Understanding of prognosis in non-metastatic prostate cancer: a randomised comparative study of clinician estimates measured against the PREDICT prostate prognostic model. *Br J Cancer* **121**, 715-718 (2019).
56. Torres, F.A., Pasarelli, I., Cutri, A., Ossorio, M.F. & Ferrero, F. Impact Assessment of a Decision Rule for Using Antibiotics in Pneumonia: A Randomized Trial. *Pediatr Pulm* **49**, 701-706 (2014).
57. True, M.W.*, et al.* Impact of a Diabetes Risk Score on Lifestyle Education and Patient Adherence (IDEA) in Prediabetes: A Multisite Randomized Controlled Trial. *Mil Med* **180**, 1091-1097 (2015).
58. van de Maat, J.S.*, et al.* Evaluation of a clinical decision rule to guide antibiotic prescription in children with suspected lower respiratory tract infection in The Netherlands: A stepped-wedge cluster randomised trial. *Plos Med* **17**(2020).
59. Van Driest, S.L.*, et al.* Acute kidney injury risk-based screening in pediatric inpatients: a pragmatic randomized trial. *Pediatr Res* **87**, 118-124 (2020).
60. de Vos-Kerkhof, E.*, et al.* Impact of a Clinical Decision Model for Febrile Children at Risk for Serious Bacterial Infections at the Emergency Department: A Randomized Controlled Trial. *Plos One* **10**(2015).
61. Voss, C.*, et al.* Effect of Wearable Digital Intervention for Improving Socialization in Children With Autism Spectrum Disorder A Randomized Clinical Trial. *Jama Pediatr* **173**, 446-454 (2019).
62. Wang, S.V.*, et al.* Stepped-wedge randomised trial to evaluate population health intervention designed to increase appropriate anticoagulation in patients with atrial fibrillation. *BMJ Qual Saf* **28**, 835-842 (2019).
63. Wijnberge, M.*, et al.* Effect of a Machine Learning-Derived Early Warning System for Intraoperative Hypotension vs Standard Care on Depth and Duration of Intraoperative Hypotension During Elective Noncardiac Surgery The HYPE Randomized Clinical Trial. *Jama-J Am Med Assoc* **323**, 1052-1060 (2020).
64. Yao, X.X.*, et al.* Artificial intelligence-enabled electrocardiograms for identification of patients with low ejection fraction: a pragmatic, randomized clinical trial. *Nat Med* **27**, 815-+ (2021).
65. Zeevi, D.*, et al.* Personalized Nutrition by Prediction of Glycemic Responses. *Cell* **163**, 1079-1094 (2015).
